# Predicting 24-hour intraocular pressure peaks and averages with machine learning

**DOI:** 10.3389/fmed.2024.1459629

**Published:** 2024-10-07

**Authors:** Ranran Chen, Jinming Lei, Yujie Liao, Yiping Jin, Xue Wang, Xiaomei Li, Danping Wu, Hong Li, Yanlong Bi, Haohao Zhu

**Affiliations:** ^1^Department of Ophthalmology, Shanghai Fifth People's Hospital, Fudan University, Shanghai, China; ^2^Software Engineering, Shenzhen Yishi Huolala Technology Company Limited, Shenzhen, China; ^3^Department of Ophthalmology, Shanghai East Hospital, School of Medicine, Tongji University, Shanghai, China; ^4^Department of Ophthalmology, Tongji Eye Institute, Tongji Hospital, School of Medicine, Tongji University, Shanghai, China

**Keywords:** intraocular pressure, 24-hour, measurement, nocturnal, machine learning, glaucoma

## Abstract

**Purpose:**

Predicting 24-hour peak and average intraocular pressure (IOP) is essential for the diagnosis and management of glaucoma. This study aimed to develop and assess a machine learning model for predicting 24-hour peak and average IOP, leveraging advanced techniques to enhance prediction accuracy. We also aimed to identify relevant features and provide insights into the prediction results to better inform clinical practice.

**Methods:**

In this retrospective study, electronic medical records from January 2014 to May 2024 were analyzed, incorporating 24-hour IOP monitoring data and patient characteristics. Predictive models based on five machine learning algorithms were trained and evaluated. Five time points (10:00 AM, 12:00 PM, 2:00 PM, 4:00 PM, and 6:00 PM) were tested to optimize prediction accuracy using their combinations. The model with the highest performance was selected, and feature importance was assessed using Shapley Additive Explanations.

**Results:**

This study included data from 517 patients (1,034 eyes). For predicting 24-hour peak IOP, the Random Forest Regression (RFR) model utilizing IOP values at 10:00 AM, 12:00 PM, 2:00 PM, and 4:00 PM achieved optimal performance: MSE 5.248, RMSE 2.291, MAE 1.694, and R^2^ 0.823. For predicting 24-hour average IOP, the RFR model using IOP values at 10:00 AM, 12:00 PM, 4:00 PM, and 6:00 PM performed best: MSE 1.374, RMSE 1.172, MAE 0.869, and R^2^ 0.918.

**Conclusion:**

The study developed machine learning models that predict 24-hour peak and average IOP. Specific time point combinations and the RFR algorithm were identified, which improved the accuracy of predicting 24-hour peak and average intraocular pressure. These findings provide the potential for more effective management and treatment strategies for glaucoma patients.

## 1 Introduction

Glaucoma, a leading cause of global blindness (1–3), underscores intraocular pressure (IOP) as the primary modifiable risk factor in its diagnosis and progression, as evidenced by numerous randomized clinical trials (4–7). IOP exhibits significant variation over the 24-hour cycle (8–10), making reliance on office-hour measurements insufficient for accurately characterizing its dynamics in glaucoma patients (11, 12). Studies have shown that nearly two-thirds of patients experience peak IOP outside of regular clinic hours, often occurring nocturnally (8–10). Therefore, comprehensive assessment of IOP parameters-including average, peak, and fluctuations-before and after interventions is crucial for evaluating therapy efficacy.

Traditional methods for 24-hour IOP monitoring typically involve hospitalization and spaced measurements, presenting logistical challenges such as increased patient burden, heightened medical workload, and limited feasibility in routine clinical settings. In 2009, Leonardi et al. (13) introduced a disposable contact lens sensor (CLS) enabling continuous IOP monitoring (Sensimed AG, Lausanne, Switzerland). However, this technology provides IOP readings in arbitrary units rather than millimeters of mercury (mmHg), hindering direct comparison with standard measurements (14). Zhang et al. (15) recently reported continuous 24-hour IOP monitoring in Chinese adults using mmHg, offering new insights into ocular physiology (15). Despite advancements in monitoring devices, these innovations exacerbate patient burdens, inflate medical costs, and restrict widespread adoption.

Previous studies, such as those conducted by Mosaed et al. (16), Fogagnolo et al. (17), and Leonardo et al. (18) have significantly advanced our understanding of the relationship between daytime and nighttime intraocular pressure (IOP) for predicting 24-hour IOP dynamics. The findings underscore the potential utility of using daytime IOP measurements to estimate nocturnal IOP peaks. However, traditional linear analysis methods may not fully capture the intricate nonlinear relationships inherent in IOP fluctuations, which could limit the accuracy and reliability of predictions.

Machine learning represents a subset of artificial intelligence that enables computer systems to learn and improve performance autonomously through data and algorithms, without explicit programming. The integration of machine learning technologies in our research provides robust tools for more precise prediction and analysis of complex IOP dynamics. Unlike traditional linear methods, machine learning algorithms excel in capturing complex nonlinear relationships, thereby enhancing the accuracy and reliability of IOP predictions.

This study aims to enhance the prediction accuracy of 24-hour peak and average intraocular pressure (IOP) using advanced machine learning algorithms. Data were collected from patients diagnosed with glaucoma suspects (GS), primary angle-closure glaucoma (PACG), and primary open-angle glaucoma (POAG) treated at the Fifth People’s Hospital of Shanghai, affiliated with Fudan University, between January 2014 and May 2024. The study explores the relationships between daytime IOP, sex, age, central corneal thickness (CCT), body mass index (BMI), blood pressure, ocular perfusion pressure, spherical equivalent (SE), use of glaucoma drugs, and 24-hour peak and average IOP.

Various combinations of five daytime IOP measurement time points-10:00 AM, 12:00 PM, 2:00 PM, 4:00 PM, and 6:00 PM-were systematically evaluated to identify the optimal subset for maintaining high predictive accuracy. Rigorous evaluations of five machine learning algorithms were conducted to determine the most effective combination of algorithms and features.

The best-performing model was selected based on these evaluations. Feature importance was assessed using Shapley Additive Explanations to provide detailed insights into each feature’s contribution to predictive accuracy. The study aims to develop a machine learning prediction model capable of accurately forecasting 24-hour peak and average IOP, thereby improving the efficiency and effectiveness of glaucoma management strategies.

## 2 Materials and methods

### 2.1 Data collection

Electronic medical records (EMRs) were gathered, containing 24-hour IOP monitoring data and basic patient demographics such as sex, age, central corneal thickness (CCT), and blood pressure. Inclusion criteria comprised patients aged 18 to 85 years, encompassing both sex, diagnosed with GS, PACG, or POAG according to the American Academy of Ophthalmology guidelines (19–21). Exclusion criteria encompassed patients who underwent 24-hour IOP monitoring in only one eye, had a history of ophthalmic surgery, other types of glaucoma, or concurrent corneal diseases.

### 2.2 Ethical statement

This study adheres to the principles outlined in the Helsinki Declaration and has received approval from the Ethics Committee of Shanghai Fifth People’s Hospital, affiliated with Fudan University (Ethics Approval No. 083). Given the retrospective nature of the study, the requirement for informed consent was waived.

### 2.3 Data preprocessing

#### 2.3.1 Dataset formation

The 24-hour IOP monitoring was conducted under inpatient conditions, commencing at 10:00 AM on the first day and continuing with measurements taken every 2 h until 8:00 AM the following day. Specific measurement times were as follows: 10:00 AM, 12:00 PM (noon), 2:00 PM, 4:00 PM, 6:00 PM, 8:00 PM, 10:00 PM, 12:00 AM (midnight), 2:00 AM, 4:00 AM, 6:00 AM, and 8:00 AM. Initiating monitoring at 10:00 AM ensured the capture of a complete 24-hour cycle of IOP data within a standard clinical schedule, effectively covering both daytime and nighttime periods. Each eye underwent a minimum of three measurements, and if the fluctuation in eye pressure among these measurements did not exceed 3 mmHg, the average value was computed for the respective time point.

Systolic blood pressure (SBP) and diastolic blood pressure (DBP) were measured using a brachial Mercury sphygmomanometer (GB3053-1993) on the upper left arm, with subjects seated for at least 3 min prior to measurement. Nursing staff expertly recorded parameters such as age, sex, height, weight, and blood pressure at 10:00 AM following patient admission.

The mean arterial pressure (MAP) was calculated using the formula: MAP = DBP + 1/3 (SBP - DBP). The mean ocular perfusion pressure (MOPP) was determined by the equation: MOPP = 2/3 (MAP - IOP). The systolic ocular perfusion pressure (SOPP) was calculated as SOPP = SBP - IOP, and the diastolic ocular perfusion pressure (DOPP) was calculated as DOPP = DBP - IOP. In these formulas, MAP, SBP, and DBP represent the mean arterial pressure, systolic blood pressure, and diastolic blood pressure, respectively, while IOP denotes the intraocular pressure. Throughout the study, blood pressure readings were consistently taken at 10:00 AM during the patients’ hospitalization. MOPP, SOPP and DOPP were calculated using the recorded blood pressure value at 10:00 AM and the simultaneous IOP measurement.

Intraocular pressure (IOP) was measured using non-contact tonometry (CT-80A, Topcon, Japan) with patients in a seated position. Central corneal thickness (CCT) was measured using a non-contact specular microscope (SP-3000P, Topcon Corporation, Tokyo, Japan). Refractive error (RE) was assessed with an automated vision tester (TOPCON, CV-5000), and the cup-to-disc (C/D) ratio was evaluated using an optical coherence tomography scanner (Cirrus OCT 4000). Intraocular pressure (IOP) values, RE, C/D ratio, and central corneal thickness (CCT) measurements were conducted by experienced physicians. Each parameter was measured three times per instance, with the average value recorded for analysis.

The dataset was partitioned into two subsets: 80% of the data served as the training set, while the remaining 20% was reserved for testing to evaluate model accuracy. To prevent data leakage and ensure robust model development in our machine learning workflow, we randomly divided the dataset into training and testing sets at the individual level, with 80% allocated for training and 20% for testing.

#### 2.3.2 Filling the missing items

In our study, a total of 96 instances of missing CCT (central corneal thickness) data were identified. We addressed these missing values by using mean imputation of the feature.

#### 2.3.3 Data normalization

Standardization was implemented to ensure consistency in feature scales, aiding in algorithm optimization for improved convergence and model performance. Sex was encoded into numerical values, streamlining data processing for algorithms and eliminating the necessity for extra conversion or preprocessing steps. Specifically, Sex was represented as 1 for males and 2 for females.

### 2.4 Feature description and selection

Through an extensive literature review, we identified key factors that may influence the onset and progression of glaucoma. Considering practicality in data collection, we carefully selected the following variables for our study: age (22–24), sex (25–27), CCT (28–31), C/D ratio (23, 31), blood pressure (32–34), ocular perfusion pressure (6, 35–37), usage details of anti-glaucoma eye drops (including number and duration), myopic refractive error (RE) (38, 39), Body Mass Index (BMI) (40, 41), and IOP measurements taken between 10 AM and 4 PM.

It is important to note that for our investigation, blood pressure and ocular perfusion pressure were specifically recorded at 10 AM, ensuring consistency in these assessments.

From our review, it became evident that studies have utilized varying time points for monitoring IOP (8–10, 16–18). To enhance predictive accuracy while minimizing the number of time points considered, we systematically investigated multiple combinations of IOP values at specific intervals throughout the day. These intervals included 10:00 AM, 12:00 PM, 2:00 PM, 4:00 PM, and 6:00 PM, covering different phases of the 24-hour period. These combinations were categorized into three main groups:

Group A (Three time points):

– A1 (10:00 AM, 12:00 PM, 2:00 PM).
– A2 (10:00 AM, 12:00 PM, 4:00 PM).
– A3 (10:00 AM, 12:00 PM, 6:00 PM).
– A4 (10:00 AM, 2:00 PM, 4:00 PM).
– A5 (10:00 AM, 2:00 PM, 6:00 PM).
– A6 (10:00 AM, 4:00 PM, 4:00 PM).
– A7 (12:00 PM, 2:00 PM, 4:00 PM).
– A8 (12:00 PM, 2:00 PM, 6:00 PM).
– A9 (12:00 PM, 4:00 PM, 6:00 PM).
– A10 (2:00 PM, 4:00 PM, 6:00 PM).

Group B (Four time points):

– B1 (10:00 AM, 12:00 PM, 2:00 PM, 4:00 PM).
– B2 (10:00 AM, 12:00 PM, 2:00 PM, 6:00 PM).
– B3 (10:00 AM, 12:00 PM, 4:00 PM, 6:00 PM).
– B4 (10:00 AM, 2:00 PM, 4:00 PM, 6:00 PM).
– B5 (12:00 PM, 2:00 PM, 4:00 PM, 6:00 PM).

Group C (Five time points):

– C (10:00 AM, 12:00 PM, 2:00 PM, 4:00 PM, 6:00 PM).

The correlation between each feature and both 24-hour peak IOP and average IOP was assessed, with a specific focus on selecting features demonstrating statistically significant relationships with these target variables (*p* < 0.05).

### 2.5 Model construction and methodology

This study employed five different machine learning algorithms: Logistic Regression (LR), Nearest Neighbors Regression (NNR), Random Forest Regression (RFR), Support Vector Regression (SVR) and K-Nearest Neighbors Regression (KNN). These algorithms were chosen to comprehensively evaluate their performance in predicting intraocular pressure, aiming to identify the most suitable models for achieving the study’s objectives. Through comparative analysis of their predictive capabilities, we aimed to understand their strengths and limitations in intraocular pressure prediction and select the optimal model to enhance prediction accuracy.

**Figure 1:**
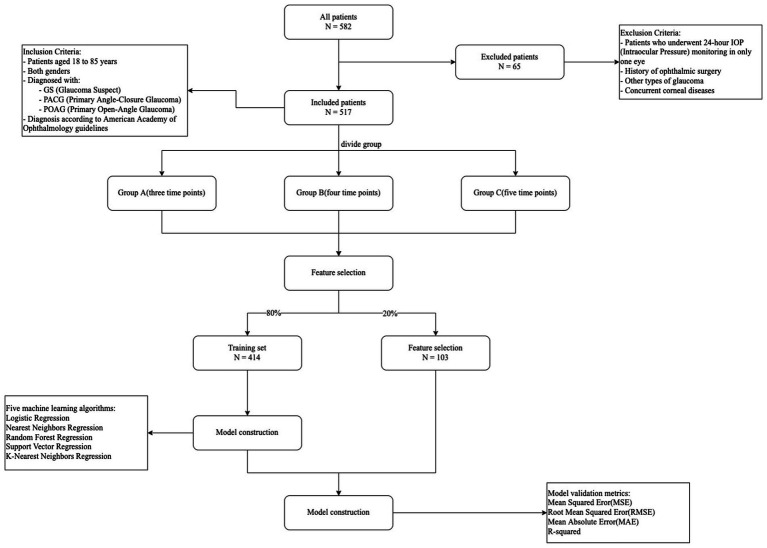
Flow of model construction.

The model training process for predicting 24-hour peak and average intraocular pressure, as depicted in Figure 1, began with retrieving electronic medical records of 582 patients who underwent 24-hour intraocular pressure monitoring at the Fifth People’s Hospital of Fudan University between January 2014 and May 2024. After applying inclusion and exclusion criteria, data from 65 patients were excluded, leaving a dataset of 514 patients for model construction. Our study aims to explore how different combinations of time points (10:00 AM, 12:00 PM, 2:00 PM, 4:00 PM, and 6:00 PM) impact prediction models. To ensure precision, we employ combinations of three, four, and five time points to construct our predictive models. The intraocular pressure readings were categorized into three groups based on combinations of measurement time points: Group A included 10 different combinations of any three time points, Group B consisted of 5 different combinations of any four time points, and Group C encompassed a single combination of all five time points. The dataset was then split into a training set (80% of the data) and a testing set (20% of the data). Five algorithms were utilized to develop predictive models using the training data. Subsequently, the performance of these models was evaluated using the testing set, and evaluation metrics were obtained to gauge their predictive accuracy.

### 2.6 Model evaluation

The performance evaluation of machine learning models typically involves multiple parameters and metrics, which contribute to assessing the model’s accuracy, generalization ability, and stability. In this study, the machine learning evaluation parameters focused on are Mean Squared Error (MSE), Root Mean Squared Error (RMSE), Mean Absolute Error (MAE), and R-squared (R^2^). These evaluation parameters provide a comprehensive assessment of the model’s performance in regression tasks, offering insights into its predictive capabilities for the target variable.

### 2.7 Statistical analysis

Sex data and the number of anti-glaucoma drugs used are discrete variables; therefore, Spearman correlation was used to analyze their relationship with the 24-hour average and peak IOP. Continuous variables such as IOP values, blood pressure, ocular perfusion pressure, age, C/D ratio, BMI, and CCT were analyzed using Pearson correlation. The correlation analysis was conducted using the Scipy library in Python. The model training and evaluation were carried out using the Sklearn library in Python.

## 3 Results

### 3.1 Demographic characteristics and data distribution of the training and testing sets

The demographic characteristics of the Overall Data, the Training Set and the Testing Set are demonstrated in Table 1. Data from 517 patients (1,034 eyes) were included in the study, obtained from electronic medical records. In this study, daytime IOP refers to measurements taken between 10:00 AM and 6:00 PM. Additionally, during our data preprocessing, we performed imputation for 96 missing corneal thickness data points.

**Table 1:** Demographic and clinical information of the patients on the study.

Characteristics	Overall data	Training set	Testing set
Number of eyes	1,034	828	206
Number of participants	517	414	103
Mean age, y(SD)	51.62 ± 16.86	51.85 ± 17.05	50.66 ± 16.03
Male–female subjects, n (%)	300/217, (58.03/41.97)	240/174, (57.97/42.03)	60/43, (58.25/41.75)
C/D ratio	0.62 ± 0.17	0.63 ± 0.19	0.58 ± 0.18
Mean CCT (um)(SD)	529.2 ± 34.07	527.56 ± 33.83	535.78.92 ± 35.51
Mean BMI (kg/m^2^), (SD)	23.54 ± 2.85	23.61 ± 2.61	23.26 ± 3.23
Mean RE(D), (SD)	−2.52 ± 3.26	−2.50 ± 3.29	−2.61 ± 3.22
Diagnosis by eye, *n* (%)			
Glaucoma suspect	626, (60.54)	500, (60.39)	126, (61.16)
Primary open-angle glaucoma	320, (30.95)	259, (31.28)	61, (29.61)
Primary angle-closure glaucoma	88, (8.51)	69, (8.33)	19, (9.23)
Number of drugs by eye, *n* (%)			
None, *n* (%)	715, (69.15)	577, (69.69)	138, (66.99)
1, *n* (%)	230, (22.24)	183, (22.1)	47, (22.81)
2, *n* (%)	79, (7.64)	61,(7.36)	18, (8.73)
3, *n* (%)	8, (0.78)	5, (0.61)	3, (1.47)
4, *n* (%)	2, (0.19)	2, (0.24)	0, (0)
Mean duration of drugs(y), (SD)	0.32 ± 0.75	0.34 ± 0.77	0.24 ± 0.69
Mean IOP10(mmHg)(SD)	18.49 ± 4.69	18.38 ± 4.76	18.93 ± 4.40
Mean IOP12(mmHg)(SD)	18.23 ± 4.70	18.13 ± 4.74	18.62 ± 4.55
Mean IOP14(mmHg)(SD)	17.75 ± 4.67	17.66 ± 4.73	18.09 ± 4.42
Mean IOP16(mmHg)(SD)	17.78 ± 4.69	17.72 ± 4.76	18.02 ± 4.40
Mean IOP18(mmHg)(SD)	17.67 ± 4.58	17.62 ± 4.6	17.87 ± 4.48
24-hour peak IOP (mmHg)(SD)	22.17 ± 5.67	22.08 ± 5.76	22.54 ± 5.33
24-hour average IOP (mmHg)(SD)	18.24 ± 4.34	18.15 ± 4.41	18.60 ± 4.05
24-fluctuation IOP (mmHg)(SD)	7.04 ± 3.12	7.01 ± 3.12	7.16 ± 3.14
Mean SBP10am(mmHg)(SD)	121.4 ± 15.18	121.79 ± 15.28	119.83 ± 14.79
Mean DBP10am(mmHg)(SD)	75.32 ± 10.04	75.4 ± 10.28	75.00 ± 9.08
Mean MAP10am(mmHg)(SD)	90.68 ± 10.45	90.87 ± 10.72	89.94 ± 9.38
Mean SOPP10am(mmHg)(SD)	102.90 ± 15.67	103.6 ± 15.98	100.09 ± 14.42
Mean DOPP10am(mmHg)(SD)	56.82 ± 10.80	57.01 ± 10.92	56.07 ± 10.32
Mean MOPP10am(mmHg)(SD)	41.85 ± 7.99	42.05 ± 8.04	41.07 ± 7.79

Variables in Table 1 include ‘y’ for year, ‘n’ for number, ‘D’ for diopters, ‘number of drugs’ indicating the count of anti-glaucoma medications used, and ‘duration of drugs’ specifying the duration of administration for these medications.

### 3.2 Analysis of 24-hour intraocular pressure distribution using box plots

**Figure 2:**
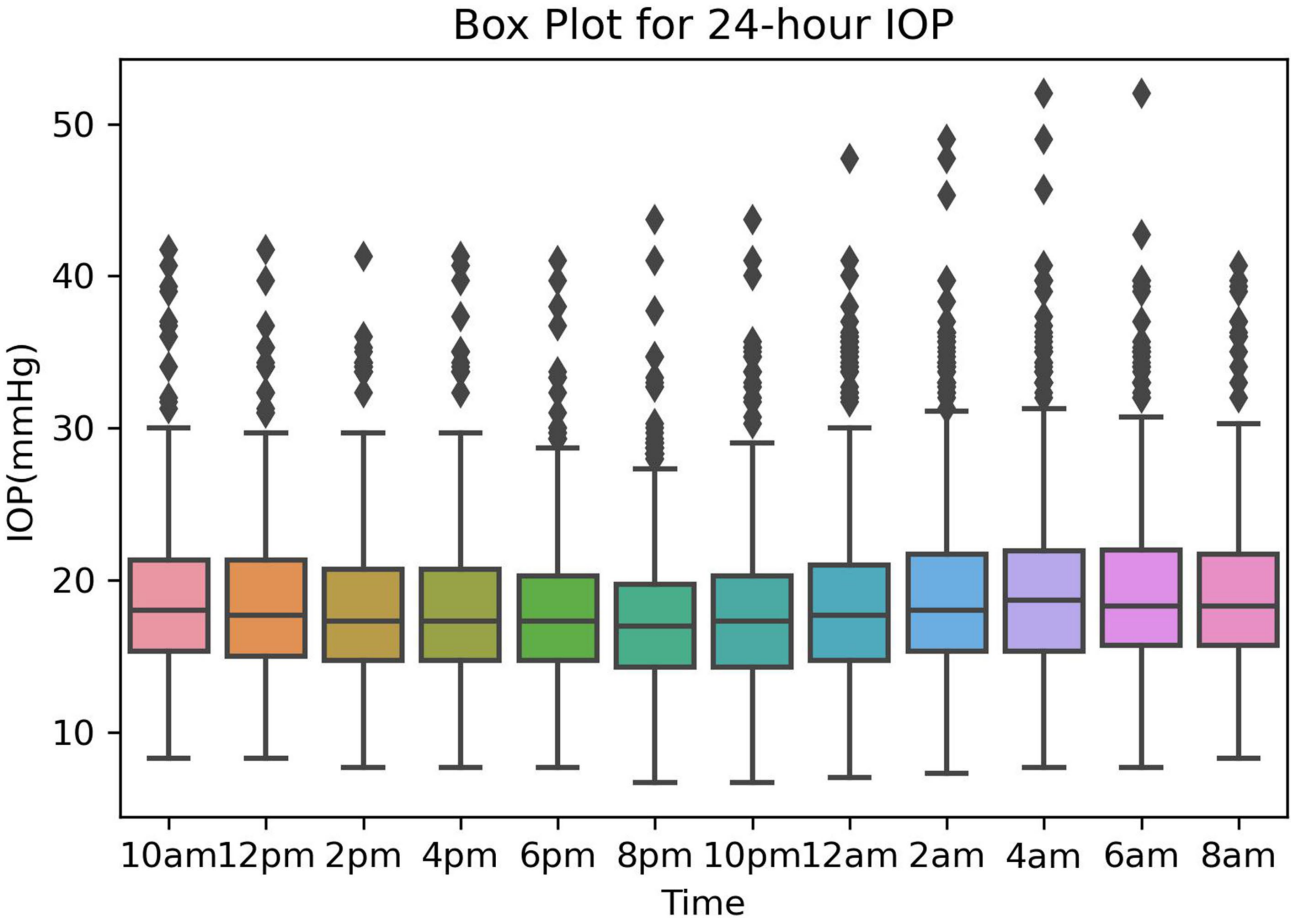
The box plot for 24-hour IOP.

The box plot for 24-hour IOP is presented in Figure 2, showing the 24-hour IOP data from the training and validation sets measured every 2 h, starting from 10:00 AM on the first day until 8:00 AM on the second day, resulting in a total of 12 IOP readings. The box plot illustrates the distribution of intraocular pressure (IOP) values at different time points. The central line within each box represents the median IOP, with the upper and lower boundaries indicating the third quartile (Q3) and first quartile (Q1), respectively, and the length of the box representing the range of IOP distribution (Figure 2).

From the box plot, it is evident that the lowest levels of IOP, including maximum, median, and minimum values, occur at 8:00 PM and 10:00 PM. Conversely, the highest IOP levels are observed at 2:00 AM and 4:00 AM. Additionally, notably high outlier values are apparent between midnight and 6:00 AM, highlighting significant IOP fluctuations during these hours.

### 3.3 Correlation analysis of features

The results of the correlation analysis between various features and the 24-hour peak IOP are presented in Table 2. The analysis indicates that sex, C/D ratio, MOPP10am, SOPP10am, and DOPP10am are negatively correlated with 24-hour average IOP, with statistical significance (*p* < 0.05). BMI, DBP10am, CCT, IOP10am, IOP2pm, IOP4pm, and IOP6pm show a positive correlation with 24-hour peak IOP, also statistically significant (*p* < 0.05). However, age, RE, duration of anti-glaucoma drug use, number of anti-glaucoma drugs used, SBP10am, and MAP10am exhibit no statistically significant correlation with 24-hour peak IOP. Based on these results, these 12 features (*p* < 0.05) were utilized in constructing the predictive model for 24-hour peak IOP.

**Table 2:** Correlation analysis results of factors and 24-hour peak IOP.

Feature	Correlation	*p* Value
Age	−0.050	0.119
Sex	−0.188	<0.0001^*^
BMI	0.134	<0.0001^*^
RE	0.051	0.115
Duration of drugs	−0.048	0.139
Number of drugs	−0.023	0.478
SBP10am	0.012	0.710
DBP10am	0.066	0.040^*^
MAP10am	0.048	0.140
CCT	0.435	<0.0001^*^
C/D ratio	−0.141	<0.0001^*^
IOP10am	0.781	<0.0001^*^
IOP12pm	0.806	<0.0001^*^
IOP2pm	0.825	<0.0001^*^
IOP4pm	0.842	<0.0001^*^
IOP6pm	0.837	<0.0001^*^
MOPP10am	−0.423	<0.0001^*^
SOPP10am	−0.238	<0.0001^*^
DOPP10am	−0.296	<0.0001^*^

The results of the correlation analysis between various features and the 24-hour average IOP are presented in Table 3. The analysis indicates that sex, the number of anti-glaucoma drugs used, C/D ratio, MOPP10am, SOPP10am, and DOPP10am are negatively correlated with 24-hour average IOP, with statistical significance (*p* < 0.05). BMI, CCT, IOP10am, IOP2pm, IOP4pm, and IOP6pm show a positive correlation with 24-hour average IOP, also statistically significant (*p* < 0.05). However, age, RE, duration of anti-glaucoma drug use, SBP10am, DBP10am, and MAP10am exhibit no statistically significant correlation with 24-hour average IOP. Based on these findings, these 12 features (*p* < 0.05) were used in developing the predictive model for 24-hour average IOP.

**Table 3:** Correlation analysis results of factors and 24-hour average IOP.

Feature	Correlation	*p* Value
Age	−0.045	0.168
Sex	−0.158	<0.0001^*^
BMI	0.143	<0.0001^*^
RE	0.009	0.771
Duration of drugs	−0.033	0.305
Number of drugs	−0.076	0.019^*^
SBP10am	−0.006	0.849
DBP10am	0.041	0.200
MAP10am	0.023	0.472
CCT	0.482	<0.0001^*^
C/D ratio	−0.187	<0.0001^*^
IOP10am	0.853	<0.0001^*^
IOP12pm	0.876	<0.0001^*^
IOP2pm	0.901	<0.0001^*^
IOP4pm	0.914	<0.0001^*^
IOP6pm	0.916	<0.0001^*^
MOPP10am	−0.486	<0.0001^*^
SOPP10am	−0.278	<0.0001^*^
DOPP10am	−0.352	<0.0001^*^

“*” indicates results with *p* < 0.05, “Number of drugs” represents the count of anti-glaucoma medications used, and “duration of drugs” indicates the duration of administration for these medications in Tables 2, 3.

### 3.4 Comparative performance of five algorithms based on different time point combinations

The performance of the 24-hour peak IOP prediction models using the best-performing time point combinations in Groups A, B, and C across five algorithms is illustrated in Table 4. The optimal combinations were A5 and A8 for Group A, B2 for Group B, and C for Group C. The combination B2 (10:00 AM, 12:00 PM, 2:00 PM, and 6:00 PM) using the RFR algorithm demonstrated the highest overall performance in predicting 24-hour peak IOP, achieving an MSE of 5.248, RMSE of 2.291, MAE of 1.694, and R^2^ value of 0.823.

**Table 4:** Performance of five algorithms in predicting peak IOP: best time points from groups A, B, and C.

Performance metrics	LR	NNR	RFR	SVR	KNN
A5 (10:00 AM, 2:00 PM, 6:00 PM)					
MSE	7.032	8.628	5.905	7.807	8.724
RMSE	2.652	2.937	2.430	2.794	2.954
MAE	1.988	2.196	1.824	1.955	2.229
R^2^	0.762	0.708	0.800	0.736	0.705
A8 (12:00 PM, 2:00 PM, 6:00 PM)					
MSE	6.880	6.878	5.920	7.621	7.943
RMSE	2.623	2.623	2.433	2.761	2.818
MAE	1.952	1.938	1.808	1.894	2.144
R^2^	0.767	0.768	0.800	0.742	0.732
B2 (10:00 AM, 12:00 PM, 2:00 PM, 6:00 PM)					
MSE	6.881	9.993	**5.248**	7.708	7.563
RMSE	2.623	3.161	**2,291**	2.776	2.75
MAE	1.952	2.786	**1.694**	1.896	2.092
R^2^	0.767	0.662	**0.823**	0.739	0.744
C (10:00 AM, 12:00 PM, 2:00 PM, 4:00 PM, 6:00 PM)					
MSE	6.758	7.496	5.925	7.289	7.134
RMSE	2.600	2.738	2.434	2.7	2.671
MAE	1.974	1.908	1.776	1.861	2.000
R^2^	0.772	0.747	0.800	0.754	0.759

LR, logistic regression; NNR, neural network regression; RFR, random forest regression; SVR, support vector regression; and KNN, k-nearest neighbors. MSE, mean squared error; RMSE, root mean squared error; MAE, mean absolute error; and R^2^, R-squared.

The performance of the 24-hour average IOP prediction models using the best-performing time point combinations in Groups A, B, and C across five algorithms is illustrated in Table 5. The optimal combinations were A8 for Group A, B3 for Group B, and C for Group C. The combination B3 (10:00 AM, 12:00 PM, 4:00 PM, and 6:00 PM) using the RFR algorithm demonstrated the highest overall performance in predicting 24-hour average IOP, achieving an MSE of 1.374, RMSE of 1.172, MAE of 0.869, and R^2^ value of 0.918.

**Table 5:** Performance of five algorithms in predicting average IOP: best time points from groups A, B, and C.

Performance metrics	LR	NNR	RFR	SVR	KNN
A8 (12:00 PM, 2:00 PM, 6:00 PM)					
MSE	1.76	2.116	1.476	1.819	3.144
RMSE	1.327	1.455	1.215	1.349	1.773
MAE	1.006	1.093	0.919	1.019	1.385
R^2^	0.895	0.874	0.912	0.892	0.813
B3 (10:00 AM, 12:00 PM, 4:00 PM, 6:00 PM)					
MSE	1.547	2.726	**1.374**	1.602	2.891
RMSE	1.244	1.651	**1.172**	1.266	1.7
MAE	0.955	1.318	**0.869**	0.965	1.306
R^2^	0.908	0.838	**0.918**	0.905	0.828
C (10:00 AM, 12:00 PM, 2:00 PM, 4:00 PM, 6:00 PM)					
MSE	1.459	1.99	1.413	1.471	2.432
RMSE	1.208	1.411	1.189	1.213	1.559
MAE	0.961	1.123	0.929	0.951	1.232
R^2^	0.913	0.882	0.916	0.913	0.855

LR, logistic regression; NNR, neural network regression; RFR, random forest regression; SVR, support vector regression; and KNN, k-nearest neighbors. MSE, mean squared error; RMSE, root mean squared error; MAE, mean absolute error; and R^2^, R-squared.

Summarizing the results of Tables 4, 5, the RFR algorithm consistently outperformed other algorithms in predicting both the 24-hour peak IOP and average IOP. Time point combination B2 demonstrated the highest values for peak IOP, while combination B3 exhibited the highest values for average IOP.

### 3.5 Scatter plots and residual plots for optimal time point combinations and algorithms

**Figure 3:**
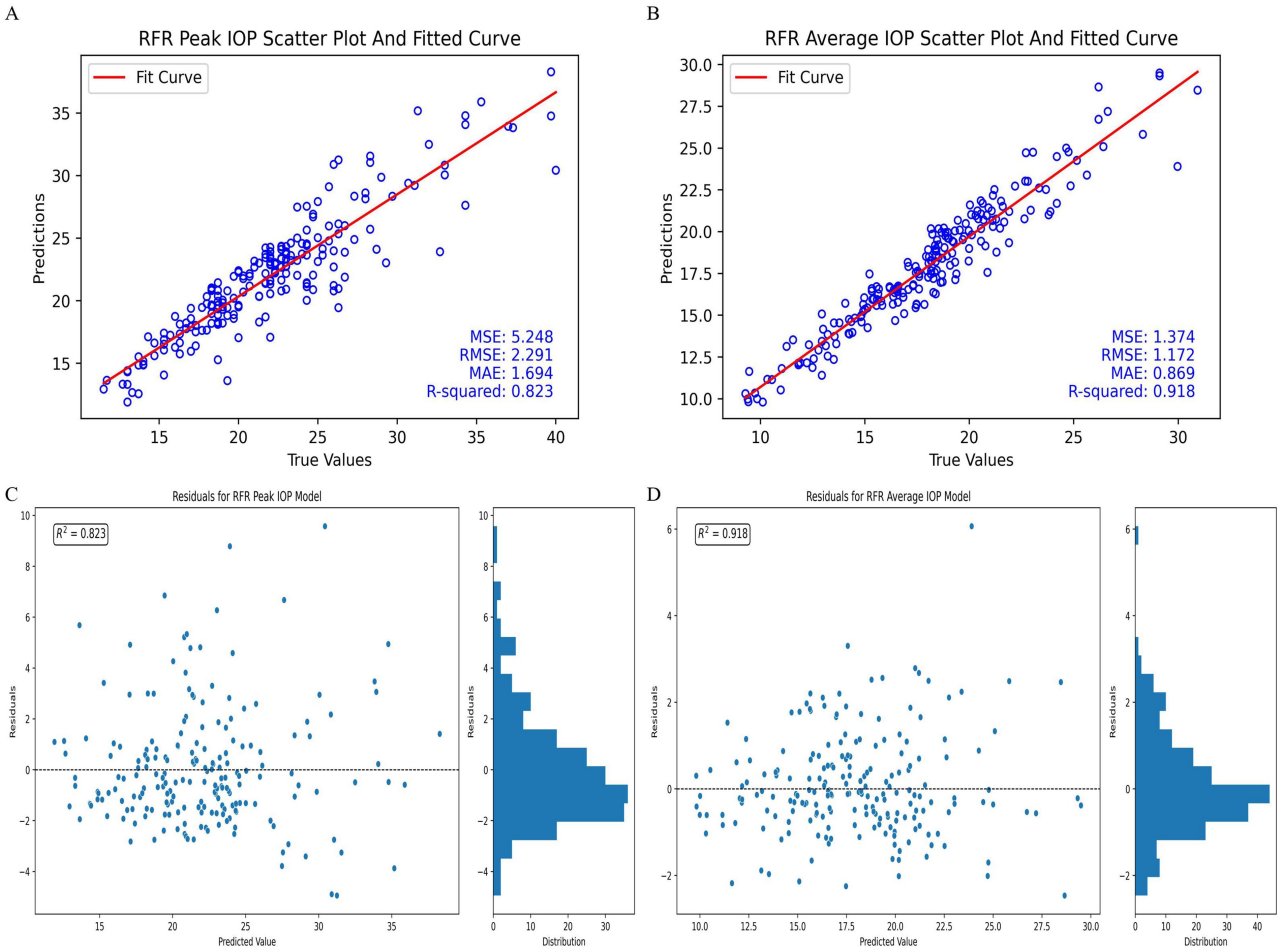
Performance of the best-performing models in predicting 24-hour IOP. **(A)** Peak IOP scatter plot and fitted curve for time point combination B2 with RFR model. **(B)** Average IOP scatter plot and fitted curve for time point combination B3 with RFR model. **(C)** Residuals for peak IOP model using time point combination B2 with RFR model. **(D)** Residuals for average IOP model using time point combination B3 with RFR model.

The performance of models with the best time point combinations for predicting IOP, both utilizing the RFR algorithm, is illustrated in Figure 3. The scatter plot for the RFR model with time point combination B2, which predicts 24-hour peak IOP, is shown, as illustrated in Figure 3A. The corresponding residuals for this model are displayed in Figure 3C. The scatter plot in Figure 3A demonstrates that the predicted values are closely aligned with the actual peak IOP values, while Figure 3C indicates that the residuals are distributed around zero, with an R^2^ value of 0.823, suggesting that most residuals are near zero.

Similarly, the scatter plot for the RFR model using time point combination B3 to predict 24-hour average IOP is presented in Figure 3B. The residuals for this model are shown in Figure 3D. The scatter plot in Figure 3B reflects a close alignment between the predicted and actual average IOP values, and Figure 3D shows that the residuals are uniformly distributed around zero, with an R^2^ value of 0.918. The histogram on the right of Figure 3D further indicates that a substantial proportion of residuals are near zero.

### 3.6 Feature importance

We utilized SHAP (Shapley Additive Explanations) to assess the importance of features in predicting both 24-hour peak and average IOP. The two models employed different sets of 12 features. The analysis focused on the top-performing models, each integrating specific time point combinations with the RFR algorithm.

**Figure 4:**
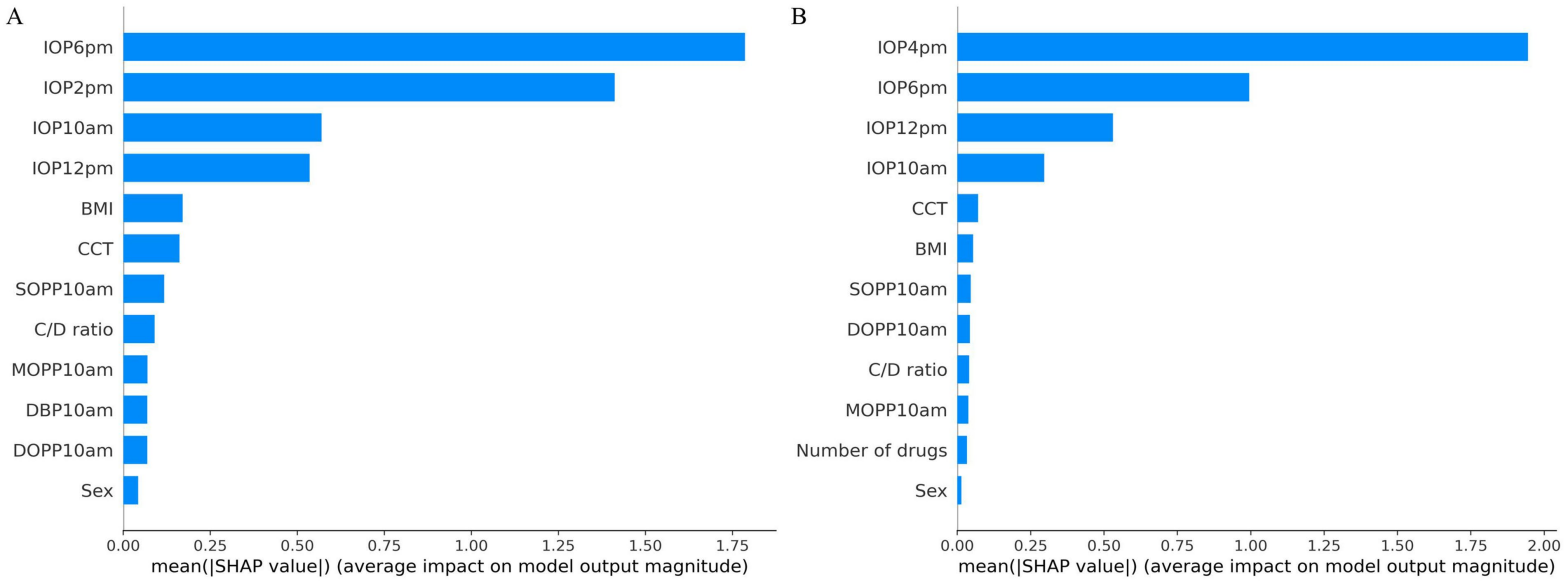
SHAP value analysis for peak and average IOP prediction models. **(A)** SHAP values for peak IOP prediction model. **(B)** SHAP values for average IOP prediction model.

The SHAP values for the model predicting 24-hour peak IOP, which employed the B2 time point combination, are illustrated in Figure 4A. Among the features, IOP measurements at 6:00 PM, 2:00 PM, 12:00 PM, and 10:00 AM demonstrated the highest average absolute SHAP values, indicating their significant predictive importance. Other features such as CCT, BMI, C/D ratio, SOPP10am, sex, MOPP10am, DBP10am, and DOPP10am had lower average SHAP values, reflecting their relatively lesser impact on the model’s predictions.

The SHAP values for the model predicting 24-hour average IOP, utilizing the B3 time point combination, are presented in Figure 4B. Among the features, IOP measurements at 4:00 PM, 6:00 PM, 12:00 PM, and 10:00 AM had the highest average absolute SHAP values, indicating their significant contribution to the model’s predictive power. Other features, including CCT, BMI, the number of drugs, MOPP10am, SOPP10am, DOPP10am, C/D ratio, and sex, also showed predictive capability but with lower average SHAP values, signifying their relatively lesser importance.

**Figure 5:**
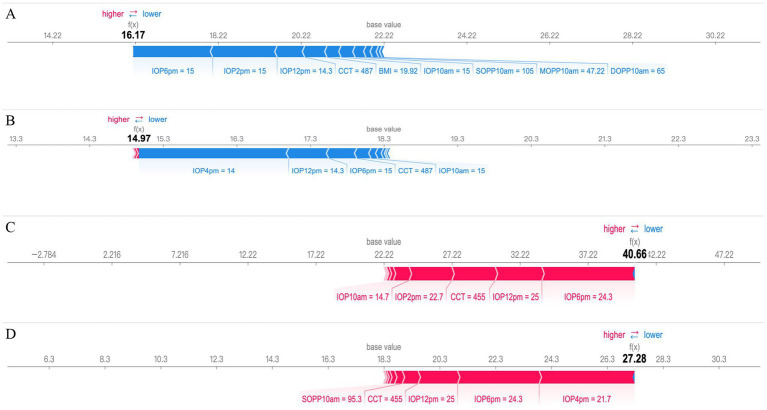
SHAP force plot interpretation of individual prediction results. **(A,B)** a case with the smallest 24-hour IOP fluctuation (1.69 mmHg) and **(C,D)** a case with the largest 24-hour IOP fluctuation (31.00 mmHg) are presented based on the SHAP force plot. In these plots, the blue bars on the right represent features that contribute to a decrease in the predicted IOP values, while the red bars on the left indicate features that lead to an increase in the predicted IOP values.

We selected two patients for analysis: one with the highest 24-hour IOP fluctuation of 31 mmHg and the other with the lowest IOP fluctuation of 1.69 mmHg. SHAP force plots, which provide individualized explanations for IOP predictions based on test set patient samples, are presented in Figures 5A–D. In these plots, the blue bars on the right represent features that contribute to lower IOP predictions, while the red bars on the left correspond to features that contribute to higher IOP predictions.

The predictions for peak and mean IOP in patients with low 24-hour IOP fluctuation, with predicted values of 16.17 and 14.97 respectively, are presented in Figures 5A,B. Key features influencing peak IOP predictions include IOP measurements at different times (such as 6:00 PM, 2:00 PM, and 12:00 PM), as well as CCT and BMI. These features are shown in blue, indicating that they collectively contribute to lower predicted IOP values. For mean IOP predictions, influential factors include IOP measurements at 4:00 PM, 12:00 PM, and 6:00 PM, along with CCT, all shown in blue, leading to lower mean IOP predictions.

The predictions for peak and mean IOP in patients with high 24-hour IOP fluctuation, with predicted values of 40.66 and 28.28, respectively, are shown in Figures 5C,D. Key factors affecting peak IOP predictions include higher IOP values at 10:00 AM, 12:00 PM, and 2:00 PM, and thinner CCT, which are displayed in red, indicating that these features lead to higher predicted IOP values. For mean IOP predictions, influential factors include higher SOPP at 10:00 AM, thinner CCT, and higher IOP measurements at 12:00 PM, 6:00 PM, and 4:00 PM, also shown in red, contributing to higher mean IOP predictions.

## 4 Discussion

### 4.1 Analysis of correlation findings

The significant correlations identified in our study highlight the complex interplay of factors influencing 24-hour peak and average intraocular pressure (IOP) in glaucoma management. Key findings include the strong correlation of specific IOP measurement times with peak and average IOP, sex differences indicating higher IOP in males (15, 25–27), and diastolic blood pressure (32–34) on IOP, and the importance of ocular perfusion pressure (6, 35–37). Additionally, anti-glaucoma medications effectively reduce average IOP, and higher BMI (40, 41) is associated with increased IOP.

Our study demonstrates a positive correlation between CCT and both 24-hour peak IOP and average IOP (*p* < 0.05). While thin CCT is commonly recognized as a risk factor for glaucoma (28–31), this finding appears contradictory to our results. This discrepancy may stem from differences in IOP measurement methods. Previous research (42) has shown that IOP values measured by NCT are generally higher than those measured by Goldmann Applanation Tonometry (GAT), with a difference of 0.95 ± 2.03 mmHg, suggesting that NCT may overestimate IOP values. Our study found that thicker CCT was associated with higher IOP measurements, which may be due to NCT’s heightened sensitivity to corneal thickness, leading to higher readings for thicker corneas. While GAT is considered the gold standard for IOP measurement (43, 44), it has certain drawbacks. GAT requires a skilled technician or ophthalmologist, along with a slit lamp, fluorescein staining, and topical anesthesia, which adds to the complexity of the procedure and increases the risk of infection. Additionally, if the examiner holds the eyelids during the measurement, this may lead to artificially elevated IOP readings. Despite these limitations, GAT remains highly reliable and widely accepted. In contrast, NCT measures IOP using an air puff, which avoids direct contact with the cornea, thereby reducing the risk of infection and eliminating the need for anesthesia and staining. Our retrospective study employing NCT revealed that it is significantly influenced by CCT, indicating that special attention should be given to CCT factors when using NCT to measure IOP to minimize measurement errors. Future research should further explore the comparative effectiveness of different measurement methods and how to optimize IOP measurement in clinical settings to enhance early glaucoma diagnosis and management.

Studies indicate that a larger C/D ratio is a significant risk factor for disease progression in primary open-angle glaucoma (23, 31). Our research found a negative correlation between the C/D ratio and both 24-hour peak IOP and average IOP. This result supports Stewart et al. (45), who linked lower average treated IOP levels with progressive optic disc damage. This may be explained by the fact that patients with a larger C/D ratio often present with more advanced glaucoma and are consequently treated with a greater number of anti-glaucoma medications, which can result in lower IOP due to more aggressive therapeutic interventions.

Although age is recognized as a major risk factor for glaucoma (22–24), our study did not demonstrate a significant correlation between age and 24-hour peak or average IOP. We hypothesize that this may be due to selection bias in our sample population. In our dataset, younger individuals were predominantly glaucoma suspects who had not yet been diagnosed or initiated IOP-lowering treatment, potentially leading to greater IOP variability. In contrast, older individuals were mostly diagnosed glaucoma patients already receiving treatment, resulting in more stable IOP levels. These differences in cohort characteristics may have confounded the potential association between age and IOP. Future studies could address this limitation by including a larger and more balanced sample of both untreated and treated glaucoma patients, which may provide a clearer understanding of the true relationship between age and IOP.

Research has indicated that for each diopter (D) increase in myopia, the risk of glaucoma increases by approximately 20%, with a notably non-linear relationship observed, especially in cases of high myopia (46). However, our study did not find a significant correlation between RE and 24-hour peak IOP or average IOP (*p* < 0.05). This result may be attributed to the characteristics of our sample, particularly the relatively low proportion of patients with high myopia. Given the non-linear nature of myopia’s impact on glaucoma risk, future research should further explore the effects of varying degrees of myopia on IOP and glaucoma risk.

### 4.2 Algorithm selection for IOP prediction

In our model training, we evaluated five algorithms-Logistic Regression (LR), Neural Network Regression (NNR), Random Forest Regression (RFR), Support Vector Regression (SVR), and k-Nearest Neighbors Regression (KNN)-to assess various data features and patterns. Each algorithm has distinct processing methods and assumptions that capture different aspects of the data. By comparing their performances, we aimed to identify the most effective algorithm for improving the accuracy and stability of 24-hour IOP predictions.

Our results indicate that RFR outperformed the other algorithms in predicting both 24-hour peak and average IOP. This performance highlights RFR’s effectiveness in capturing the complexities of IOP fluctuations throughout the day. This performance is likely due to RFR’s ensemble learning approach, which reduces overfitting by combining multiple decision trees and enhances prediction accuracy. Additionally, RFR handles nonlinear relationships and high-dimensional data effectively, demonstrating stability and robustness.

### 4.3 Optimal time point combinations for predicting 24-hour IOP

Considering that using multiple time points to predict 24-hour intraocular pressure (IOP) can enhance prediction accuracy, it also increases clinical costs and reduces convenience for patients and doctors. Conversely, choosing too few time points may result in insufficient data, thereby affecting the accuracy of 24-hour IOP predictions. Therefore, this study selected five time points (10:00 AM, 12:00 PM, 2:00 PM, 4:00 PM, and 6:00 PM) for IOP measurements and explored various combinations of these time points.

In predicting 24-hour peak IOP, the combination of 10:00 AM, 12:00 PM, 2:00 PM, and 6:00 PM (B2) performed the best. For predicting 24-hour average IOP, the combination of 10:00 AM, 12:00 PM, 4:00 PM, and 6:00 PM (B3) also yielded the optimal results. These findings suggest that selecting four well-distributed time points can provide the best balance between prediction accuracy and clinical feasibility.

### 4.4 Interpretation of feature importance using SHAP in prediction models

In this study, we employed SHAP to evaluate the importance of the best models for predicting 24-hour peak IOP and average IOP, as shown in Figures 4A,B. We also selected cases with the smallest and largest 24-hour IOP variability to further interpret the predictions for peak and average IOP using SHAP, as illustrated in Figures 5A–D. The results demonstrate that measurements of IOP at specific time points significantly impact the prediction of 24-hour IOP, while other features have relatively minor effects. Based on these findings, future research should explore additional relevant features and consider employing alternative machine learning models to enhance predictive performance. Furthermore, investigating the physiological mechanisms underlying the significance of specific IOP measurement times will contribute to a deeper understanding of glaucoma pathophysiology.

### 4.5 Limitations

Despite our positive findings, our study has several limitations. Firstly, our data sources and sample scope are limited, requiring broader data validation and external verification to ensure the model’s universality and reliability. Additionally, while the Goldmann applanation tonometer (GAT) is widely regarded as the standard for IOP measurement (43, 44), our retrospective study used the CT-80 non-contact tonometer (NCT) for 24-hour IOP monitoring. NCT may be influenced by corneal characteristics such as thickness and elasticity, potentially leading to an overestimation of IOP, especially in patients with thicker corneas (23, 29). Therefore, future research should focus on improving measurement methods to enhance the accuracy of IOP assessments and better understand disease progression. Additionally, regarding nocturnal IOP monitoring, Weber et al. observed that supine IOP is typically higher than seated IOP due to postural changes affecting fluid dynamics (47). Since our study measured nocturnal IOP in the seated position, future research should enhance IOP monitoring methodologies to better understand nocturnal IOP fluctuations. We also appreciate the feedback on the feasibility of implementing machine learning models for predicting 24-hour peak and average IOP in clinical settings. We fully recognize that obtaining multiple IOP measurements throughout the day is not practical in routine outpatient environments due to constraints such as time, manpower, and resources. Typically, only 1–2 IOP measurements are obtained during a clinic visit, which limits the direct applicability of models requiring more frequent measurements. Despite these challenges, our study represents an initial step toward bridging the gap between current clinical practices and the potential for more comprehensive IOP monitoring.

## 5 Conclusion

Our study has developed machine learning models capable of relatively accurate predictions for 24-hour peak and average IOP. These models may offer new insights into glaucoma management. We have made progress in balancing cost with prediction accuracy while enhancing the interpretability and applicability of the models. To further improve the clinical utility of these models, future work should focus on identifying the most representative 1–2 IOP measurement time points and integrating additional clinical features, such as visual field test results and retinal nerve fiber layer (RNFL) data, to enhance the models’ predictive power and clinical value. Additionally, exploring the integration of these models with home monitoring devices represents an important direction for improving practical application. Home-based tonometers could enable patients to measure their IOP multiple times a day, potentially reducing the need for frequent clinic visits. Combining machine learning models with these home monitoring tools may provide physicians with more comprehensive daily IOP data, which could assist in optimizing treatment decisions.

## Data Availability

The original contributions presented in the study are included in the article/Supplementary material, further inquiries can be directed to the corresponding authors.

## Supplementary material

Due to space constraints, we have abbreviated the results regarding the prediction models for 24-hour peak and average intraocular pressure (IOP) using different time point combinations (Groups A, B, and C) and five machine learning algorithms. We have retained in the main text only the best-performing time point combinations from each group across the five algorithms. The complete dataset is provided in the Supplementary Table S1 for 24-hour peak IOP and Supplementary Table S2 for average IOP predictions.
