# Corrigendum: Predicting 24-hour intraocular pressure peaks and averages with machine learning

**DOI:** 10.3389/fmed.2024.1513862

**Published:** 2024-11-08

**Authors:** Ranran Chen, Jinming Lei, Yujie Liao, Yiping Jin, Xue Wang, Xiaomei Li, Danping Wu, Hong Li, Yanlong Bi, Haohao Zhu

**Affiliations:** ^1^Department of Ophthalmology, Shanghai Fifth People's Hospital, Fudan University, Shanghai, China; ^2^Software Engineering, Shenzhen Yishi Huolala Technology Company Limited, Shenzhen, China; ^3^Department of Ophthalmology, Shanghai East Hospital, School of Medicine, Tongji University, Shanghai, China; ^4^Department of Ophthalmology, Tongji Eye Institute, Tongji Hospital, School of Medicine, Tongji University, Shanghai, China

**Keywords:** intraocular pressure, 24-hour, measurement, nocturnal, machine learning, glaucoma

In the published article, there was an error in Figure 4. During the final confirmation process, we updated the term “gender” to “sex” in the manuscript; however, we regrettably overlooked this change in Figure 4. The corrected Figure 4 and its caption appear below.

**Figure 4:**
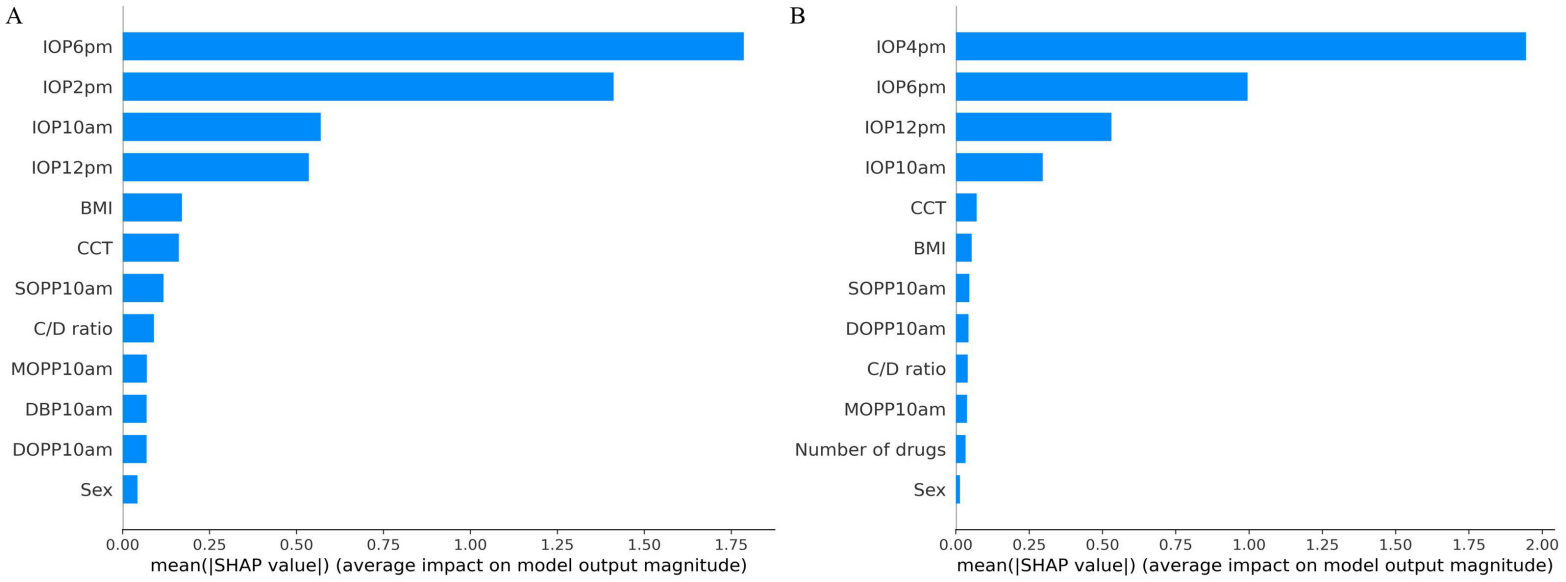
SHAP value analysis for peak and average IOP prediction models. **(A)** SHAP values for peak IOP prediction model. **(B)** SHAP values for average IOP prediction model.

The authors apologize for this error and state that this does not change the scientific conclusions of the article in any way. The original article has been updated.

