# Supplementary material for: Predicting 24-hour intraocular pressure peaks and averages with machine learning
Source: Front Med (Lausanne). 2024 Oct 7;11:1459629. doi: 10.3389/fmed.2024.1459629 (PMC11493148; doi:10.3389/fmed.2024.1459629)
Supplement: Supplementary file 1 [file Table_1.DOCX]

Supplementary Material

# Supplementary Tables

The performance of 24-hour peak intraocular pressure (IOP) and average IOP prediction models using time point combinations from groups A, B, and C, under five different algorithms, is illustrated in Supplementary Tables 1 and 2.

## Supplementary Table 1

The performance of 24-hour peak IOP prediction models using time point combinations from groups A, B, and C, under five different algorithms, is illustrated in Supplementary Tables 1. The B2 combination (10:00 AM, 12:00 PM, 2:00 PM, 6:00 PM) with the RFR algorithm demonstrated the best performance for the 24-hour peak IOP prediction model, achieving an MSE of 5.248, an RMSE of 2.291, an MAE of 1.694, and an R² of 0.823.

SUPPLEMENTARY TABLE 1 Performance of five algorithms in predicting peak IOP across different time point combinations

| A1 (10:00 AM, 12:00 PM, 2:00 PM) | | | | | |
| --- | --- | --- | --- | --- | --- |
| Performance metrics | LR | NNR | RFR | SVR | KNN |
| MSE | 7.235 | 7.519 | 6.044 | 7.823 | 8.755 |
| RMSE | 2.690 | 2.742 | 2.458 | 2.797 | 2.959 |
| MAE | 2.088 | 2.055 | 1.836 | 1.982 | 2.229 |
| R^2^ | 0.755 | 0.746 | 0.796 | 0.736 | 0.704 |
| A2 (10:00 AM, 12:00 PM, 4:00 PM) | | | | | |
| Performance metrics | LR | NNR | RFR | SVR | KNN |
| MSE | 7.197 | 8.382 | 6 | 7.772 | 8.508 |
| RMSE | 2.683 | 2.895 | 2.449 | 2.788 | 2.917 |
| MAE | 2.043 | 1.963 | 1.785 | 1.943 | 2.161 |
| R^2^ | 0.757 | 0.717 | 0.797 | 0.737 | 0.712 |
| A3 (10:00 AM, 12:00 PM, 6:00 PM) | | | | | |
| Performance metrics | LR | NNR | RFR | SVR | KNN |
| MSE | 7.544 | 8.204 | 6.271 | 8.412 | 9.303 |
| RMSE | 2.747 | 2.864 | 2.504 | 2.900 | 3.050 |
| MAE | 2.026 | 2.001 | 1.827 | 1.956 | 2.236 |
| R^2^ | 0.745 | 0.723 | 0.788 | 0.716 | 0.686 |
| A4 (10:00 AM, 2:00 PM, 4:00 PM) | | | | | |
| Performance metrics | LR | NNR | RFR | SVR | KNN |
| MSE | 7.348 | 7.337 | 6.409 | 7.769 | 8.764 |
| RMSE | 2.711 | 2.709 | 2.532 | 2.787 | 2.96 |
| MAE | 2.117 | 2.123 | 1.884 | 2.028 | 2.252 |
| R^2^ | 0.752 | 0.752 | 0.783 | 0.737 | 0.704 |
| A5 (10:00 AM, 2:00 PM, 6:00 PM) | | | | | |
| Performance metrics | LR | NNR | RFR | SVR | KNN |
| MSE | 7.032 | 8.628 | 5.905 | 7.807 | 8.724 |
| RMSE | 2.652 | 2.937 | 2.430 | 2.794 | 2.954 |
| MAE | 1.988 | 2.196 | 1.824 | 1.955 | 2.229 |
| R^2^ | 0.762 | 0.708 | 0.800 | 0.736 | 0.705 |
| A6 (10:00 AM, 4:00 PM, 6:00 PM) | | | | | |
| Performance metrics | LR | NNR | RFR | SVR | KNN |
| MSE | 7.277 | 15.233 | 6.205 | 8.036 | 8.868 |
| RMSE | 2.698 | 3.903 | 2.491 | 2.835 | 2.978 |
| MAE | 2.043 | 3.525 | 1.824 | 1.969 | 2.234 |
| R^2^ | 0.754 | 0.485 | 0.790 | 0.728 | 0.700 |
| A7 (12:00 PM, 2:00 PM, 4:00 PM) | | | | | |
| Performance metrics | LR | NNR | RFR | SVR | KNN |
| MSE | 7.036 | 9.589 | 6.594 | 7.304 | 7.849 |
| RMSE | 2.653 | 3.097 | 2.568 | 2.703 | 2.802 |
| MAE | 2.069 | 2.691 | 1.916 | 1.899 | 2.143 |
| R^2^ | 0.762 | 0.676 | 0.777 | 0.753 | 0.735 |
| A8 (12:00 PM, 2:00 PM, 6:00 PM) | | | | | |
| Performance metrics | LR | NNR | RFR | SVR | KNN |
| MSE | 6.880 | 6.878 | 5.920 | 7.621 | 7.943 |
| RMSE | 2.623 | 2.623 | 2.433 | 2.761 | 2.818 |
| MAE | 1.952 | 1.938 | 1.808 | 1.894 | 2.144 |
| R^2^ | 0.767 | 0.768 | 0.800 | 0.742 | 0.732 |
| A9 (12:00 PM, 4:00 PM, 6:00 PM) | | | | | |
| Performance metrics | LR | NNR | RFR | SVR | KNN |
| MSE | 6.938 | 19.344 | 6.339 | 7.636 | 8.591 |
| RMSE | 2.634 | 4.398 | 2.518 | 2.763 | 2.931 |
| MAE | 1.978 | 3.506 | 1.813 | 1.881 | 2.179 |
| R^2^ | 0.765 | 0.346 | 0.786 | 0.742 | 0.710 |
| A10 (2:00 PM, 4:00 PM, 6:00 PM) | | | | | |
| Performance metrics | LR | NNR | RFR | SVR | KNN |
| MSE | 6.868 | 7.292 | 6.688 | 7.393 | 8.164 |
| RMSE | 2.621 | 2.700 | 2.586 | 2.719 | 2.857 |
| MAE | 1.997 | 1.997 | 1.916 | 1.911 | 2.201 |
| R^2^ | 0.768 | 0.754 | 0.774 | 0.750 | 0.724 |
| B1 (10:00 AM, 12:00 PM, 2:00 PM, 4:00 PM) | | | | | |
| Performance metrics | LR | NNR | RFR | SVR | KNN |
| MSE | 7.035 | 7.439 | 6.133 | 7.286 | 7.507 |
| RMSE | 2.652 | 2.728 | 2.476 | 2.699 | 2.74 |
| MAE | 2.068 | 1.912 | 1.809 | 1.9 | 2.085 |
| R^2^ | 0.762 | 0.749 | 0.793 | 0.754 | 0.746 |
| B2 (10:00 AM, 12:00 PM, 2:00 PM, 6:00 PM) | | | | | |
| Performance metrics | LR | NNR | **RFR** | SVR | KNN |
| MSE | 6.881 | 9.993 | **5.248** | 7.708 | 7.563 |
| RMSE | 2.623 | 3.161 | **2291** | 2.776 | 2.75 |
| MAE | 1.952 | 2.786 | **1.694** | 1.896 | 2.092 |
| R^2^ | 0.767 | 0.662 | **0.823** | 0.739 | 0.744 |
| B3 (10:00 AM, 12:00 PM, 4:00 PM, 6:00 PM) | | | | | |
| Performance metrics | LR | NNR | RFR | SVR | KNN |
| MSE | 6.938 | 8.393 | 5.797 | 7.551 | 8.133 |
| RMSE | 2.634 | 2.897 | 2.408 | 2.748 | 2.852 |
| MAE | 1.979 | 2.061 | 1.731 | 1.87 | 2.079 |
| R^2^ | 0.766 | 0.716 | 0.804 | 0.745 | 0.725 |
| B4 (10:00 AM, 2:00 PM, 4:00 PM, 6:00 PM) | | | | | |
| Performance metrics | LR | NNR | RFR | SVR | KNN |
| MSE | 6.868 | 7.037 | 6.238 | 7.32 | 7.914 |
| RMSE | 2.621 | 2.653 | 2.498 | 2.706 | 2.813 |
| MAE | 1.997 | 1.933 | 1.849 | 1.915 | 2.114 |
| R^2^ | 0.768 | 0.762 | 0.789 | 0.753 | 0.732 |
| B5 (12:00 PM, 2:00 PM, 4:00 PM, 6:00 PM) | | | | | |
| Performance metrics | LR | NNR | RFR | SVR | KNN |
| MSE | 6.757 | 6.976 | 6.2 | 7.3 | 7.32 |
| RMSE | 2.599 | 2.641 | 2.49 | 2.702 | 2.706 |
| MAE | 1.975 | 1.827 | 1.84 | 1.862 | 2.035 |
| R^2^ | 0.772 | 0.764 | 0.790 | 0.753 | 0.753 |
| C (10:00 AM, 12:00 PM, 2:00 PM, 4:00 PM, 6:00 PM) | | | | | |
| Performance metrics | LR | NNR | RFR | SVR | KNN |
| MSE | 6.758 | 7.496 | 5.925 | 7.289 | 7.134 |
| RMSE | 2.600 | 2.738 | 2.434 | 2.7 | 2.671 |
| MAE | 1.974 | 1.908 | 1.776 | 1.861 | 2.000 |
| R^2^ | 0.772 | 0.747 | 0.800 | 0.754 | 0.759 |

## Supplementary table 2

The performance of 24-hour average IOP prediction models using time point combinations from groups A, B, and C, under five different algorithms, is illustrated in Supplementary Tables 2. The B3 combination (10:00 AM, 12:00 PM, 4:00 PM, 6:00 PM) with the RFR algorithm demonstrated the best performance for the 24-hour average IOP prediction model, achieving an MSE of 1.374, an RMSE of 1.172, an MAE of 0.869, and an R² of 0.918.

SUPPLEMENTARY TABLE 2 Performance of five algorithms in predicting average IOP across different time point combinations

| A1 (10:00 AM, 12:00 PM, 2:00 PM) | | | | | |
| --- | --- | --- | --- | --- | --- |
| Performance metrics | LR | NNR | RFR | SVR | KNN |
| MSE | 2.075 | 2.094 | 1.94 | 2.043 | 3.306 |
| RMSE | 1.441 | 1.447 | 1.393 | 1.429 | 1.818 |
| MAE | 1.176 | 1.166 | 1.093 | 1.152 | 1.439 |
| R^2^ | 0.877 | 0.876 | 0.885 | 0.879 | 0.803 |
| A2 (10:00 AM, 12:00 PM, 4:00 PM) | | | | | |
| Performance metrics | LR | NNR | RFR | SVR | KNN |
| MSE | 1.795 | 2.286 | 1.717 | 1.844 | 3.419 |
| RMSE | 1.34 | 1.512 | 1.31 | 1.358 | 1.849 |
| MAE | 1.06 | 1.163 | 0.994 | 1.055 | 1.421 |
| R^2^ | 0.893 | 0.864 | 0.898 | 0.89 | 0.797 |
| A3 (10:00 AM, 12:00 PM, 6:00 PM) | | | | | |
| Performance metrics | LR | NNR | RFR | SVR | KNN |
| MSE | 2.003 | 3.434 | 1.71 | 2.076 | 3.68 |
| RMSE | 1.415 | 1.853 | 1.308 | 1.441 | 1.918 |
| MAE | 1.075 | 1.43 | 0.979 | 1.085 | 1.463 |
| R^2^ | 0.881 | 0.796 | 0.898 | 0.877 | 0.781 |
| A4 (10:00 AM, 2:00 PM, 4:00 PM) | | | | | |
| Performance metrics | LR | NNR | RFR | SVR | KNN |
| MSE | 1.954 | 2.049 | 1.806 | 1.916 | 3.207 |
| RMSE | 1.398 | 1.431 | 1.344 | 1.384 | 1.791 |
| MAE | 1.135 | 1.132 | 1.085 | 1.124 | 1.426 |
| R^2^ | 0.884 | 0.878 | 0.893 | 0.886 | 0.809 |
| A5 (10:00 AM, 2:00 PM, 6:00 PM) | | | | | |
| Performance metrics | LR | NNR | RFR | SVR | KNN |
| MSE | 1.675 | 1.796 | 1.622 | 1.7 | 3.408 |
| RMSE | 1.294 | 1.34 | 1.273 | 1.304 | 1.846 |
| MAE | 1.022 | 1.078 | 0.977 | 1.027 | 1.462 |
| R^2^ | 0.9 | 0.893 | 0.904 | 0.899 | 0.797 |
| A6 (10:00 AM, 4:00 PM, 6:00 PM) | | | | | |
| Performance metrics | LR | NNR | RFR | SVR | KNN |
| MSE | 1.706 | 2.186 | 1.637 | 1.759 | 3.488 |
| RMSE | 1.306 | 1.478 | 1.279 | 1.326 | 1.868 |
| MAE | 1.016 | 1.19 | 0.978 | 1.018 | 1.439 |
| R^2^ | 0.899 | 0.87 | 0.903 | 0.895 | 0.793 |
| A7 (12:00 PM, 2:00 PM, 4:00 PM) | | | | | |
| Performance metrics | LR | NNR | RFR | SVR | KNN |
| MSE | 1.902 | 2.3 | 1.84 | 1.899 | 2.963 |
| RMSE | 1.379 | 1.517 | 1.356 | 1.378 | 1.721 |
| MAE | 1.089 | 1.211 | 1.064 | 1.081 | 1.354 |
| R^2^ | 0.887 | 0.863 | 0.891 | 0.887 | 0.824 |
| A8 (12:00 PM, 2:00 PM, 6:00 PM) | | | | | |
| Performance metrics | LR | NNR | RFR | SVR | KNN |
| MSE | 1.76 | 2.116 | 1.476 | 1.819 | 3.144 |
| RMSE | 1.327 | 1.455 | 1.215 | 1.349 | 1.773 |
| MAE | 1.006 | 1.093 | 0.919 | 1.019 | 1.385 |
| R^2^ | 0.895 | 0.874 | 0.912 | 0.892 | 0.813 |
| A9 (12:00 PM, 4:00 PM, 6:00 PM) | | | | | |
| Performance metrics | LR | NNR | RFR | SVR | KNN |
| MSE | 1.625 | 1.672 | 1.533 | 1.698 | 3.303 |
| RMSE | 1.275 | 1.293 | 1.238 | 1.303 | 1.817 |
| MAE | 0.982 | 0.985 | 0.925 | 0.991 | 1.408 |
| R^2^ | 0.903 | 0.901 | 0.909 | 0.899 | 0.804 |
| A10 (2:00 PM, 4:00 PM, 6:00 PM) | | | | | |
| Performance metrics | LR | NNR | RFR | SVR | KNN |
| MSE | 1.75 | 4.316 | 1.762 | 1.875 | 3.219 |
| RMSE | 1.323 | 2.077 | 1.327 | 1.369 | 1.794 |
| MAE | 1.04 | 1.8 | 1.01 | 1.062 | 1.388 |
| R^2^ | 0.896 | 0.743 | 0.895 | 0.889 | 0.809 |
| B1 (10:00 AM, 12:00 PM, 2:00 PM, 4:00 PM) | | | | | |
| Performance metrics | LR | NNR | RFR | SVR | KNN |
| MSE | 1.742 | 2.73 | 1.56 | 1.702 | 2.627 |
| RMSE | 1.32 | 1.652 | 1.249 | 1.305 | 1.621 |
| MAE | 1.063 | 1.341 | 1.002 | 1.051 | 1.285 |
| R^2^ | 0.896 | 0.838 | 0.907 | 0.899 | 0.844 |
| B2 (10:00 AM, 12:00 PM, 2:00 PM, 6:00 PM) | | | | | |
| Performance metrics | LR | NNR | **RFR** | SVR | KNN |
| MSE | 1.609 | 1.818 | 1.462 | 1.644 | 2.888 |
| RMSE | 1.268 | 1.348 | 1.209 | 1.282 | 1.7 |
| MAE | 0.985 | 1.082 | 0.929 | 0.987 | 1.332 |
| R^2^ | 0.904 | 0.892 | 0.913 | 0.902 | 0.828 |
| B3 (10:00 AM, 12:00 PM, 4:00 PM, 6:00 PM) | | | | | |
| Performance metrics | LR | NNR | RFR | SVR | KNN |
| MSE | 1.547 | 2.726 | 1.374 | 1.602 | 2.891 |
| RMSE | 1.244 | 1.651 | 1.172 | 1.266 | 1.7 |
| MAE | 0.955 | 1.318 | 0.869 | 0.965 | 1.306 |
| R^2^ | 0.908 | 0.838 | 0.918 | 0.905 | 0.828 |
| B4 (10:00 AM, 2:00 PM, 4:00 PM, 6:00 PM) | | | | | |
| Performance metrics | LR | NNR | RFR | SVR | KNN |
| MSE | 1.532 | 2.213 | 1.54 | 1.574 | 2.619 |
| RMSE | 1.238 | 1.488 | 1.241 | 1.255 | 1.618 |
| MAE | 0.991 | 1.262 | 0.967 | 0.994 | 1.287 |
| R^2^ | 0.909 | 0.868 | 0.908 | 0.906 | 0.844 |
| B5 (12:00 PM, 2:00 PM, 4:00 PM, 6:00 PM) | | | | | |
| Performance metrics | LR | NNR | RFR | SVR | KNN |
| MSE | 1.549 | 1.749 | 1.467 | 1.587 | 2.708 |
| RMSE | 1.245 | 1.323 | 1.211 | 1.26 | 1.646 |
| MAE | 0.966 | 0.997 | 0.935 | 0.97 | 1.298 |
| R^2^ | 0.908 | 0.896 | 0.913 | 0.906 | 0.839 |
| C (10:00 AM, 12:00 PM, 2:00 PM, 4:00 PM, 6:00 PM) | | | | | |
| Performance metrics | LR | NNR | RFR | SVR | KNN |
| MSE | 1.459 | 1.99 | 1.413 | 1.471 | 2.432 |
| RMSE | 1.208 | 1.411 | 1.189 | 1.213 | 1.559 |
| MAE | 0.961 | 1.123 | 0.929 | 0.951 | 1.232 |
| R^2^ | 0.913 | 0.882 | 0.916 | 0.913 | 0.855 |
