# Supplementary material for: Predicting 24-hour intraocular pressure peaks and averages with machine learning
Source: Front Med (Lausanne). 2024 Oct 7;11:1459629. doi: 10.3389/fmed.2024.1459629 (PMC11493148; doi:10.3389/fmed.2024.1459629)
Supplement: Supplementary file 2 [file Table_2.DOCX]

Supplementary Material

# Supplementary Tables

Thanks to the reviewers' valuable suggestions, we conducted a correlation analysis followed by a collinearity analysis. Factors with a variance inflation factor (VIF) greater than 10 were removed. The remaining features were then used for model training and validation. The performance of the 24-hour peak and mean IOP prediction models across five algorithms is summarized in Supplementary Tables 3 and 4.

Supplementary table 3 Performance metrics of 24-Hour peak IOP prediction models using five machine learning algorithms

| Performance metrics | LR | NNR | **RFR** | SVR | KNN |
| --- | --- | --- | --- | --- | --- |
| MSE | 6.825 | 7.268 | **5.624** | 7.264 | 5.777 |
| RMSE | 2.612 | 2.696 | **2.372** | 2.695 | 2.404 |
| MAE | 1.988 | 1.945 | **1.759** | 1.862 | 1.822 |
| R^2^ | 0.769 | 0.754 | **0.81** | 0.754 | 0.805 |

Supplementary table 4 Performance metrics of 24-Hour average IOP prediction models using five machine learning algorithms

| Performance metrics | LR | NNR | **RFR** | SVR | KNN |
| --- | --- | --- | --- | --- | --- |
| MSE | 1.589 | 2.084 | **1.562** | 1.566 | 1.751 |
| RMSE | 1.261 | 1.443 | **1.25** | 1.251 | 1.323 |
| MAE | 0.972 | 1.133 | **0.946** | 0.96 | 0.997 |
| R^2^ | 0.906 | 0.876 | **0.907** | 0.907 | 0.896 |

Given the results detailed in Supplementary Tables 3 and 4, we decided not to incorporate the collinearity analysis into the final model. This decision was based on our observation that the performance of the model did not improve compared to the previous approach using features selected with *p* <0.05.

We are open to further suggestions or feedback to refine our methodology if necessary. If you have any insights or recommendations on this matter, please feel free to contact us for additional modifications.
